# Comparability of thyroid-stimulating hormone immunoassays using fresh frozen human sera and external quality assessment data

**DOI:** 10.1371/journal.pone.0253324

**Published:** 2021-06-15

**Authors:** Shunli Zhang, Fei Cheng, Hua Wang, Jiangping Wen, Jie Zeng, Chuanbao Zhang, Wensong Liu, Ning Wang, Tingting Jia, Mo Wang, Rui Zhang, Yuhong Yue, Jing Xu, Zhanyong Wang, Yilong Li, Wenxiang Chen, Qingtao Wang

**Affiliations:** 1 Department of Clinical Laboratory, Beijing Chaoyang Hospital, Beijing Center for Clinical Laboratories, The Third Clinical Medical College of Capital Medical University, Beijing, P.R. China; 2 Department of Clinical Laboratory, Wangjing Hospital, China Academy of Traditional Chinese Medicine, Beijing, P.R. China; 3 Department of Clinical Laboratory, China-Japan Friendship Hospital, Beijing, P.R. China; 4 Department of Clinical Laboratory, Beijing Tongren Hospital, Capital Medical University, Beijing, P.R. China; 5 National Center for Clinical Laboratories, Beijing Hospital, National Center of Gerontology, Beijing Engineering Research Center of Laboratory Medicine, Beijing, P.R. China; 6 Department of Clinical Laboratory, Beijing Hospital, Beijing, P.R. China; 7 Department of Clinical Laboratory, Beijing Chaoyang Hospital, The Third Clinical Medical College of Capital Medical University, Beijing, P.R. China; Holbaek Sygehus, DENMARK

## Abstract

**Background:**

This study aimed to assess the comparability among assays using freshly frozen human sera and external quality assessment (EQA) data in China.

**Methods:**

Twenty-nine serum samples and two commercial EQA materials, obtained from the National Center for Clinical Laboratories (NCCL), were analyzed in triplicate using eight routine TSH assays. The commutability of commercial EQA materials (NCCL materials) was evaluated in accordance with the CLSI EP30-A and IFCC bias analysis. Median values obtained for the NCCL EQA materials were used to determine the systematic and commutability-related biases among immunoassays through back-calculation. The comparability of TSH measurements from a panel of clinical samples and NCCL EQA data was determined on the basis of Passing–Bablok regression. Furthermore, human serum pools were used to perform commutable EQA.

**Results:**

NCCL EQA materials displayed commutability among three or five of seven assay combinations according CLSI or IFCC approach, respectively. The mean of systematic bias ranged from -13.78% to 9.85% for the eight routine TSH assays. After correcting for systematic bias, averaged commutability-related biases ranged between -42.26% and 12.19%. After correction for systematic and commutability -related biases, the slopes indicating interassay relatedness ranged from 0.801 to 1.299 using individual human sera, from 0.735 to 1.254 using NCCL EQA data, and from 0.729 to 1.115 using pooled human serum EQA(the commutable EQA).

**Conclusions:**

The harmonization of TSH measurement is challenging; hence, systematic and commutability-related biases should be determined and corrected for accurate comparisons among assays when using human individual serum and the commercial EQA materials.

## Introduction

Thyroid disease is a global health problem that can impact well-being, particularly during pregnancy and childhood [1–3]. Although widespread thyroid function testing has resulted in a reduction in the prevalence of undiagnosed thyroid disease [4], there remains a lack of consistency in inter-laboratory results and consensus. Thyroid-stimulating hormone (TSH) has long been used to evaluate thyroid function [5], and some studies have developed robust factor analysis models in an effort to harmonize TSH measurements [6]. Moreover, in 2010, the International Federation of Clinical Chemistry Working Group for standardization of Thyroid Function Tests published three reports on the standardization of these tests [7–9].

Currently, external quality assessment (EQA) is not only an essential component of laboratory management systems but also used as an index for monitoring the status of standardization. In this regard, although commutable EQA samples, particularly human serum pools, are used to determine the same numeric relationship among different measurement procedures and laboratories expected for patient samples [10], it is difficult to obtain such commutable materials, owing to certain limitations associated with factors including concentration, quantity, and transportation [11].

In this study, we obtained data to conduct an assessment for TSH measurement, namely (1) the comparability of TSH immunoassays, for which systematic biases were corrected, using a panel of clinical patient samples in China; (2) the commutability of National Center for Clinical Laboratories (NCCL) commercial EQA materials; (3) the comparability of TSH immunoassays, for which commutability-related biases were corrected, using NCCL EQA materials; and (4) the comparability of TSH immunoassays using pools of fresh human serum. Furthermore, we established a method to determine precise relationships among TSH immunoassays based a panel of individual human serum samples and non-commutable commercial EQA materials (NCCL EQA materials).

## Materials and methods

### Serum panel and EQA materials

Twenty-nine serum samples containing different TSH concentrations were obtained from the clinical laboratory of Beijing Chaoyang Hospital, Capital Medical University (Beijing, China), after approval from its human ethics committee (document 2018-2-26-1). Each of these samples was aliquoted into eight portions and frozen at -80°C until use. All 29 specimens were non-hemolyzed and non-lipemic and contained TSH ranging from 0.09 to 84.03 μIU/mL, as determined using a Siemens ADVIA CentaurXP immunoassay (Siemens Healthineers, Tarrytown, NY, USA), collected from January 2018 to July 2018. All authors could not access to information that could identify individual participants during or after data collection. Two commercial EQA materials, which were randomly interspersed among the 29 samples of patient, were obtained from NCCL. All samples were analyzed in triplicate after internal quality controls were passed. On the one hand, the commutable EQA materials (human serum pool), which were prepared as previously described [12], but not frozen, were measured in cities in the provinces of Beijing, Tianjin, Hebei, and Shandong.

### Immunoassay platform

The eight TSH immunoassays used in this study were as follows: the ADVIA CentaurXP (Siemens Healthineers, Tarrytown, NY, USA), the Immulite 2000 (Siemens Healthineers, Gwynedd, UK), the DXI800 (Beckman Coulter, Brea, CA, USA), the Autolumo A2000plus (Autobio Diagnostics, Zhengzhou, China), the Maglumi2000plus (Snibe, Shenzhen, China), the Cobas 601 (Roche Diagnostics, Mannheim, Germany), the Architect i2000sr (Abbott Diagnostics, Abbott Park, IL, USA), and the Liaison XL (DiaSorin S.p.A, Saluggia, Italy). Each of these assays was performed using the respective manufacturer’s reagents and calibrators. Detailed information on these assays is provided in **S1 Table**.

### Data analysis

After excluding outliers on the basis of three standard deviations, the median values of the NCCL EQA materials were used to determine the systematic biases. The commutability of the EQA materials was carried out following Clinical and Laboratory Standards Institute (CLSI) guideline EP30-A and difference in bias based on the recommendations by the IFCC Working Group on Commutability [12–15] and commutability-related biases were determined on the basis of back-calculation among assays after correcting for systematic biases *via Deming regression*.

The commutability assessment was done according to linear regression analysis firstly, the log-transformed mean concentration of each serum sample was plotted vs log-transformed mean concentration obtained with ADVIA CentaurXP. The Deming regression and 95% prediction interval around this regression were plotted using formulas described in CLSI EP30-A. The log-transformed median concentrations of NCCL EQA materials were also plotted, the commutability were confirmed if its data point was inside the 95% prediction interval. The commutability-related biases were calculated with Eq 1.


$$\mathrm{Bias}(\%)=\frac{(C_{\mathrm{mean},\mathrm{other}}-(10^{\log C_{mean,cen}*\beta +\alpha }))*100}{\left(10^{\log C_{mean,cen}*\beta +\alpha }\right)}$$ (1)

Where C_mean,other_ was the measured TSH average concentration with other systems, C_mean,cen_ was the measured TSH average concentration with ADVIA CentaurXP, β and α were the slope and intercept with Deming regression, respectively.

The commutability assessment was done according to difference in bias analysis secondly,

The bias of each serum sample calculated as the difference between the ln-transformed mean concentration obtained with the other method and the ln-transformed mean concentration obtained with ADVIA CentaurXP, was plotted against mean of other method and ADVIA CentaurXP. Lines for the average bias for the human serum sample, black line, and the criteria for commutability (C) of the EQA materials, dashed lines, are shown on the plot. A C value of 0.237 (about 23.7% in concentration, based on desirable specification for total error from biologic variation of TSH) was used for this example. The uncertainty of the difference in bias between the human serum sample and the EQA material was shown for each EQA material as error bars. The uncertainty consists of two components: the uncertainty of the estimate of bias for the human serum samples and the uncertainty (substituted by the uncertainty of human serum samples because position effects could not be obtained for this study) of the estimate of bias for each EQA material. The associated expanded uncertainty was calculated with the coverage factor 1.9 multiplied the uncertainty. When the expanded uncertainty interval was inside C lines the EQA material was commutable, when it was outside C lines the EQA materialwas non-commutable.

**Fig 1:**
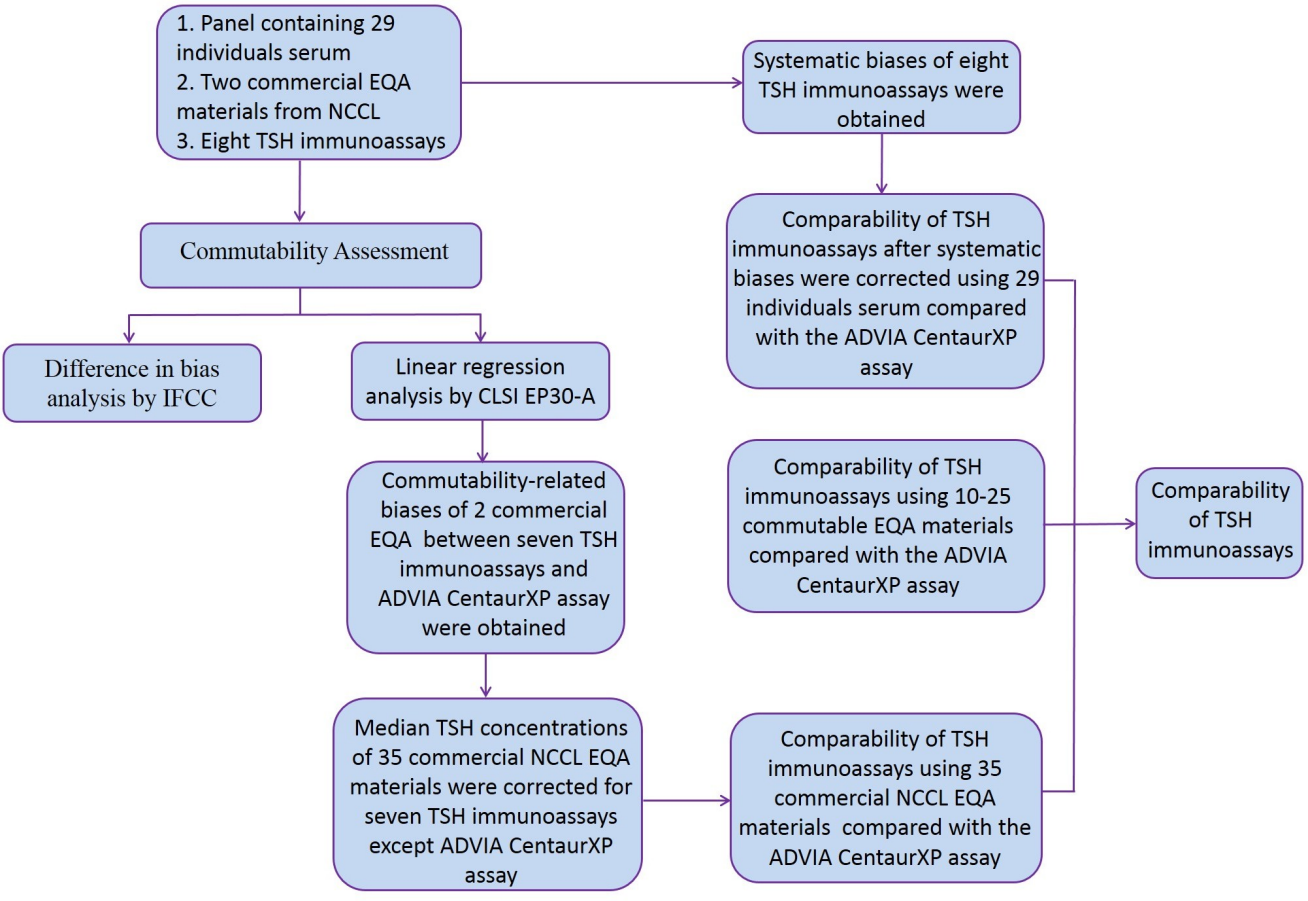
A workflow chart demonstrating the comparability of eight TSH immunoassays using three methods.

After correcting for systematic and commutability-related biases, the comparability of TSH immunoassays using individual serum samples and commercial EQA data (from 2016 to 2019) was determined via Passing–Bablok regression using MedCalc Statistical Software version 19.0.7 (MedCalc Software bvba, Ostend, Belgium). The comparability among assay measurements was also assessed using commutable EQA. Pearson correlation coefficients were also calculated. Coefficients of variation (CVs) of every EQA materials were calculated after outliers (any laboratory results that is more than 3 standard deviations) were removed in two EQA programs for these eight platforms, respectively. CV of within-laboratory was calculated from triplicate measurements. All the comparisons were based on the use of ADVIA CentaurXP as a comparative assay because this platform were always used for clinical sample measurement in our laboratory which has been accredited by International Standardization Organization (ISO) 15189 and College of American Pathologists (CAP). A workflow chart were showed in **Fig 1**.

## Results

### Descriptive statistics

**Fig 2:**
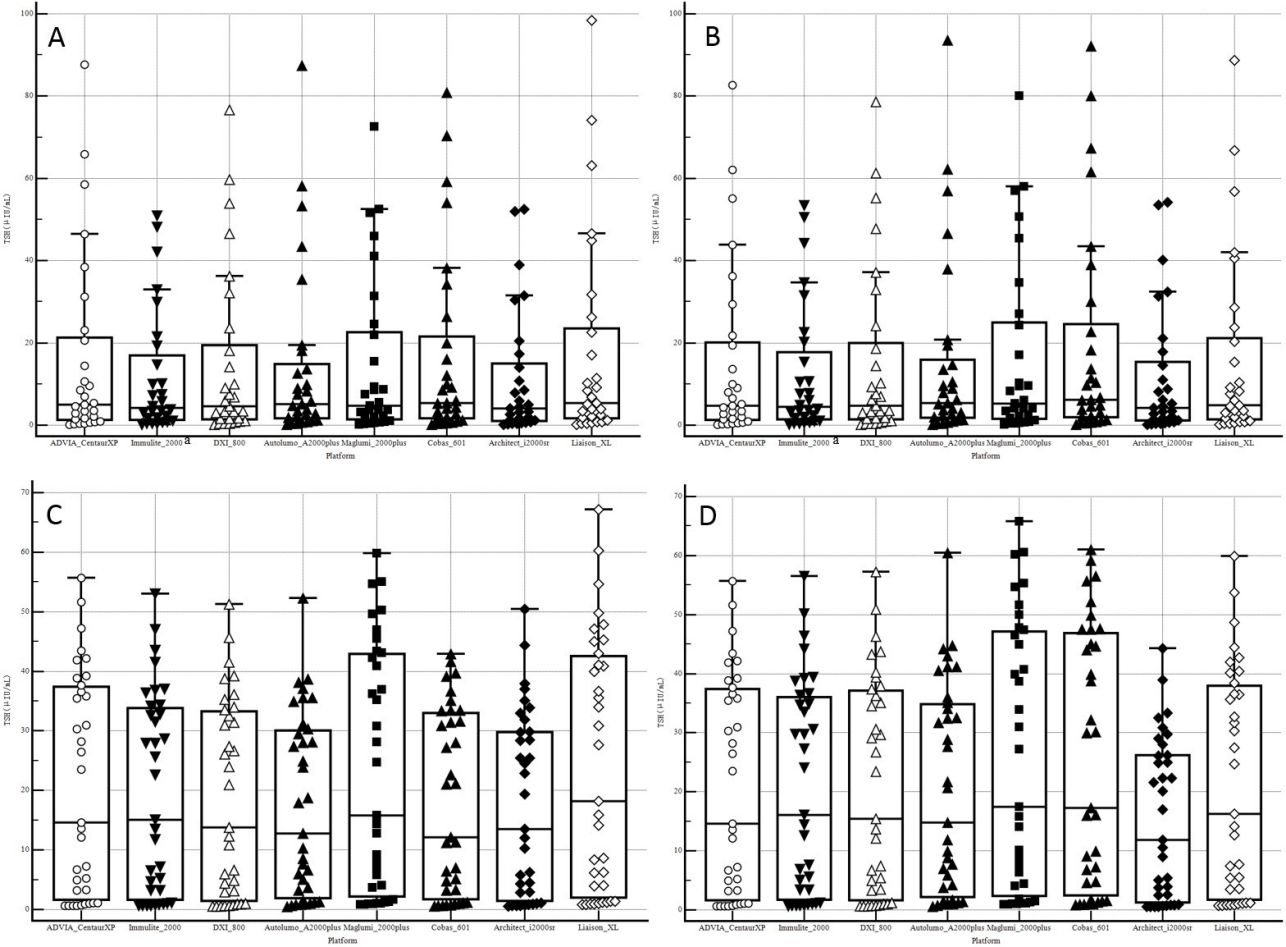
Boxplot showing the distribution of thyroid-stimulating hormone (TSH) measurement results for the eight assessed immunoassays. A and B, Results before and after systematic bias correction using individual human serum samples, respectively. C and D, Results before and after commutability-related bias correction using NCCL EQA samples, respectively. a, Results for one sample were excluded from analysis, as these were outside the analytical measurement range.

The concentrations of TSH in the 29 individual samples and EQA samples determined using eight different immunoassays are presented in **Fig 2** before and after systematic/commutability-related biases were corrected, detailed in **Tables 1** and **2**. Almost all samples were assessed thrice, with just a few samples being tested twice owing to an insufficient volume of serum obtained from one individual. Furthermore, measurements were not obtained for one sample, as the TSH concentrations exceeded the upper limit of the analytical measurement range (>75 μIU/mL) of the Immulite 2000 platform. Between 2016 and 2019, and 2017 and 2019, 1666 to 2453 laboratories participated in the NCCL EQA Program and 145 to 477 participated in the commutable EQA program, respectively. Details of the numbers of peer groups were listed in **S2** and **S3 Tables**. Similar CVs between these two EQA programs were obtained, with medians of 3.75~6.25% and 2.82~6.33%, respectively (**Tables 2** and **3**). Furthermore, using individual human sera, we obtained within-laboratory median CV values of 1.17%~4.09% (**Table 1**).

**Table 1:** Passing–Bablok regression analysis measured TSH values for patient samples after systematic biases were corrected.

Platform	sample no.	Concentration,	CV, Median(%)^2^	Regression parameters		r
		Median(range) (μIU/mL)^1^		Slope (95% CI)	Intercept, μIU/ml (95% CI)	
ADVIA CentaurXP	29	4.707(0.076–82.625)	1.82			
Immulite 2000	28	4.421(0.091–53.395)	3.07	1.033(0.956~1.107)	0.135(0.052~0.367)	0.996
DXI800	29	4.762(0.116–78.601)	2.28	1.024(0.986~1.089)	0.093(-0.005~0.226)	0.998
Autolumo A2000 plus	29	5.438(0.071–93.576)	1.36	1.049(1.026~1.110)	0.337(0.145~0.528)	0.990
Maglumi2000plus	29	5.197(0.087–80.081)	3.82	1.172(1.063~1.242)	0.093(-0.001~0.295)	0.992
Cobas 601	29	6.193(0.100–92.081)	4.09	1.299(1.222~1.371)	0.147(0.043~0.372)	0.995
Architect i2000sr	29	4.202(0.081–54.140)	1.57	0.801(0.727~0.861)	0.077(0.021~0.258)	0.990
Liaison XL	29	4.811(0.097–88.712)	1.17	1.069(1.015~1.087)	0.093(0.022~0.166)	0.999

1, Median and range of TSH concentration of patient samples were showed on eight platforms.

2, Median of coefficients of variation of TSH triplicate measurements for different patient samples were used to describe imprecisions of eight platforms.

Regression parameters were estimated between ADVIA CentaurXP and other platform. Slope and intercept were used to reveal the proportional and constant deviations between platforms.

r, denote pearson correlation coefficient. CI, denote confidence interval.

**Table 2:** Passing–Bablok regression analysis measured TSH values using NCCL EQA materials (2016~2019) after commutability-related biases were corrected.

Platform	sample no.	Concentration,	CV, Median (%)^2^	Regression parameters		
		Median (range) (μIU/mL)^1^		Slope (95% CI)	Intercept, μIU/ml (95% CI)	r
ADVIA CentaurXP^a^	35	23.485 (0.610–55.670)	4.16			
Immulite 2000^b^	35	24.087 (0.650–56.519)	6.14	0.996(0.960~1.024)	0.064(0.030~0.327)	0.997
DXI800^c^	35	23.392 (0.636–57.214)	6.25	0.997(0.979~1.015)	0.013(-0.020~0.105)	0.999
Autolumo A2000 plus^d^	35	20.693 (0.544–60.482)	5.74	0.959(0.921~1.035)	0.342(-0.041~0.784)	0.992
Maglumi2000 plus^e^	35	27.226 (0.902–65.788)	5.71	1.211(1.172~1.285)	0.164(0.063~0.324)	0.995
Cobas 601^f^	35	29.988 (0825–60.973)	3.75	1.254(1.162~1.277)	0.216(0.163~0.682)	0.995
Architect i2000sr^g^	35	17.035 (0.518–44.247)	4.67	0.735(0.707~0.757)	0.093(0.048~0.225)	0.997
Liaison XL^h^	35	24.691 (0.736–59.929)	5.93	1.034(1.022~1.047)	0.136(0.102~0.221)	0.999

a, included Siemens Advia Centaur CP/XP

b, included Siemens Immulite 2000/2000 XPi

c, included Beckman DXI 600,DXI 800

d, included AutoLumo A2000/A2000 plus

e, included Snibe Maglumi 600/800/1000/1000Plus/2000/2000plus

f, included Roche Cobas e601/e602

g, included Abbott Architect i2000SR/i2000/i1000srP

h, included DiaSorin S.p.A LIALSON/XL.

1, Median and range of TSH concentration of NCCL EQA samples were showed on eight platforms.

2, Median of coefficients of variation of TSH laboratory results for different NCCL EQA samples were used to describe imprecisions of eight platforms.

Regression parameters were estimated between ADVIA CentaurXP and other platform. Slope and intercept were used to reveal the proportional and constant deviations between platforms.

r, denote pearson correlation coefficient. CI, denote confidence interval.

**Table 3:** Passing–Bablok regression analysis measured TSH values using commutable EQA materials (2017~2019).

Platform	sample no.	Concentration	CV, Median (%)^2^	Regression parameters	
		Median (range) (μIU/mL)^1^		Slope (95% CI)	Intercept, μIU/ml (95% CI)	r
ADVIA CentaurXP^a^	25	2.98(1.32–16.80)	5.44			
Immulite 2000^b^	15	3.26(1.34–15.45)	5.55	1.065(0.976~1.141)	0.139(-0.111~0.572)	0.997
DXI800^c^	25	3.15(1.31–17.58)	6.33	1.027(1.010~1.054)	0.003(-0.075~0.089)	1.000
Autolumo A2000 plus^d^	15	3.59(1.65–16.18)	4.17	1.056(0.955~1.121)	0.284(0.053~0.596)	0.997
Maglumi2000 plus^e^	20	3.50(1.46–15.88)	4.35	1.115(1.044~1.181)	-0.149(-0.363~0.081)	0.995
Cobas 601^f^	25	3.42(1.51–18.35)	4.24	1.105(1.083~1.161)	0.072(-0.102~0.135)	1.000
Architect i2000sr^g^	25	2.36(1.04–12.25)	6.19	0.729(0.697~0.755)	0.089(0.022~0.190)	0.999
Liaison XL^h^	10	3.19(1.53–17.01)	2.82	1.111(1.083~1.151)	0.096(0.004~0.275)	1.000

a, included Siemens Advia Centaur CP/XP

b, included Siemens Siemens Immulite/One/1000/2000

c, included Beckman DXI 600,DXI 800

d, included AutoLumo A2000/A2000plus

e, included Snibe Maglumi 600/800/1000/1000Plus/2000/2000plus

f, includedRoche Cobas e601/e602

g, included Abbott Architect i2000SR/i2000/i1000sr

h, included DiaSorin S.p.A LIALSON

1, Median and range of TSH concentration of commutable EQA samples were showed on eight platforms.

2, Median of coefficients of variation of TSH laboratory results for different commutable EQA samples were used to describe imprecisions of eight platforms.

Regression parameters were estimated between ADVIA CentaurXP and other platform. Slope and intercept were used to reveal the proportional and constant deviations between platforms.

r, denote pearson correlation coefficient. CI, denote confidence interval.

### Systematic and commutability-related biases

**Fig 3:**
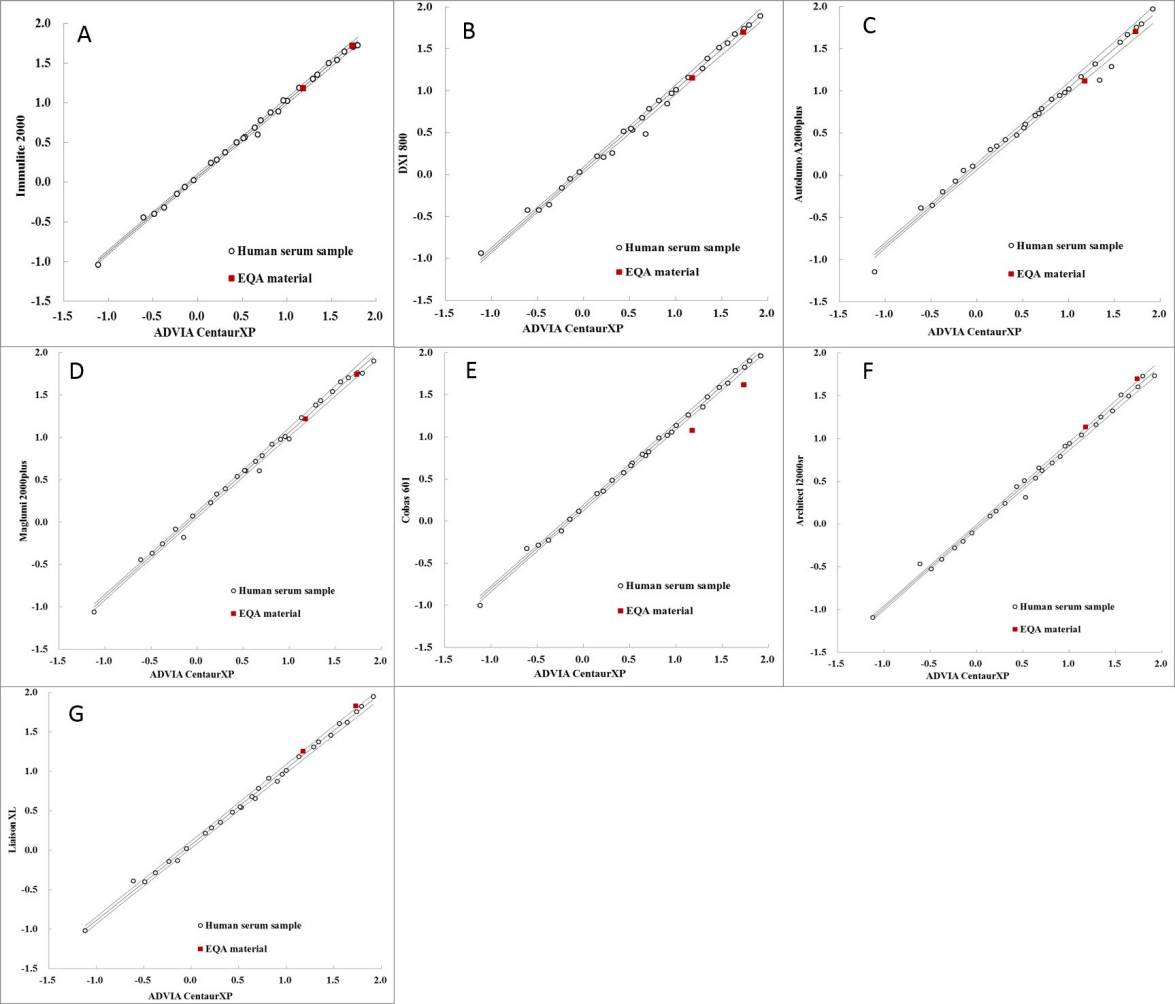
Commutability of National Center for Clinical Laboratories external quality assessment (EQA) materials among the eight assessed thyroid-stimulating hormone (TSH) instrument platforms based on CLSI approach.

**Fig 4:**
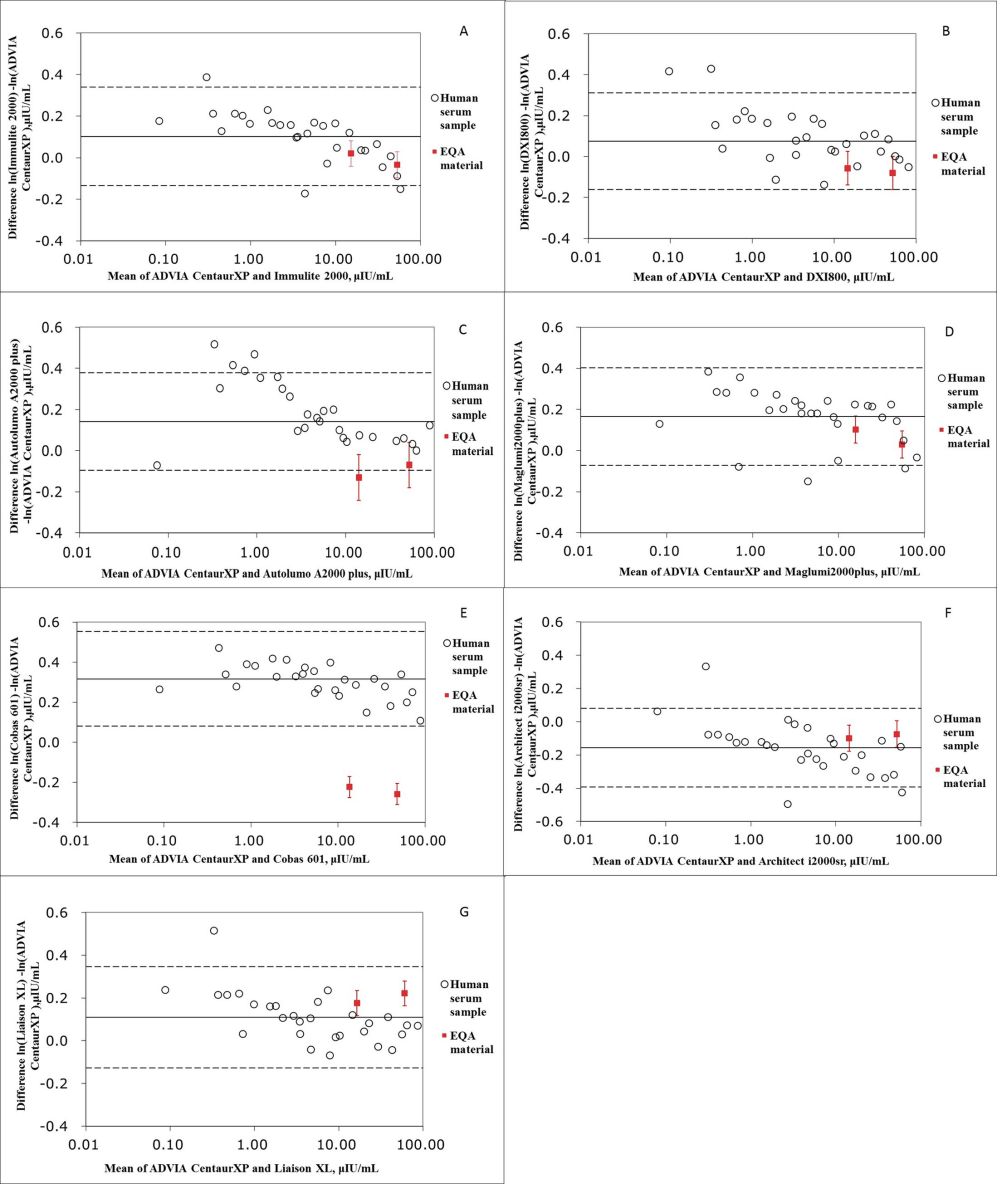
Commutability of National Center for Clinical Laboratories external quality assessment (EQA) materials among the eight assessed thyroid-stimulating hormone (TSH) instrument platforms based on IFCC approach.

Median TSH concentrations of the NCCL EQA materials obtained from 2016 to 2019 and measured using different assays were listed in **Table 2**. The means of systematic and commutability-related biases for two lot EQA materials (201811 and 201812) were -13.84%~9.85% and -38.83%~18.65%, respectively. After correcting for systematic bias, the mean commutability-related bias was between -42.26% and 12.19% (**S4** and **S5 Tables**). The commutability of materials among different assays was shown in **Fig 3**. The NCCL EQA materials were commutable for three of the seven assay pairs (**Fig 3A, 3B** and **3D**) with smaller commutability-related biases (-11.55%~-6.58%) after correcting for the systematic bias based on CLSI approach. However, these NCCL EQA materials were commutable for five of the seven assay pairs (**Fig 4A, 4B, 4D, 4F and 4G**) according to IFCC method.

### Comparability of the analytical results for TSH

Herein, we analyzed patient samples using eight assays to evaluate the comparability of different routine clinical laboratory measurement procedures after correcting for the systematic bias. The medians and ranges of the measured TSH concentrations and measurement imprecisions for the patient samples were shown in **Table 1**. Among these eight assays, compared with the ADVIA CentaurXP assay, the Cobas 601 assay displayed the greatest measurements, with a median concentration of 6.193 μIU/mL and slope (indicating interassay relatedness) of 1.299. In contrast, the Architect i2000sr assay showed lowest measurement results, with a median concentration of 4.202 μIU/mL and a slope of 0.801 (**Table 1**). Among the 29 samples analyzed, we detected a 1000-fold difference between the lowest and highest concentrations of TSH (e.g., from 0.07 to 82.625 μIU/mL using the ADVIA CentaurXP assay), and the Pearson correlation coefficients ranged from 0.990 to 0.999.

The NCCL EQA and commutable EQA data revealed similar results, with the Cobas assay yielding higher median values (29.988 μIU/mL and 3.42 μIU/mL, respectively) and larger slopes (1.254 and 1.105, respectively), and the Architect assay yielding lower medians (17.035 μIU/mL and 2.36 μIU/mL, respectively) and smaller slopes (0.735 and 0.729, respectively) (**Tables 2** and **3**). An approximately 10-fold difference was observed between the lowest and highest concentrations of TSH in the two EQA programs (e.g., NCCL EQA: between 0.610 and 55.670 μIU/mL; commutable EQA: between 1.32 and 16.80 μIU/mL, using the ADVIA CentaurXP assay).

**Fig 5:**
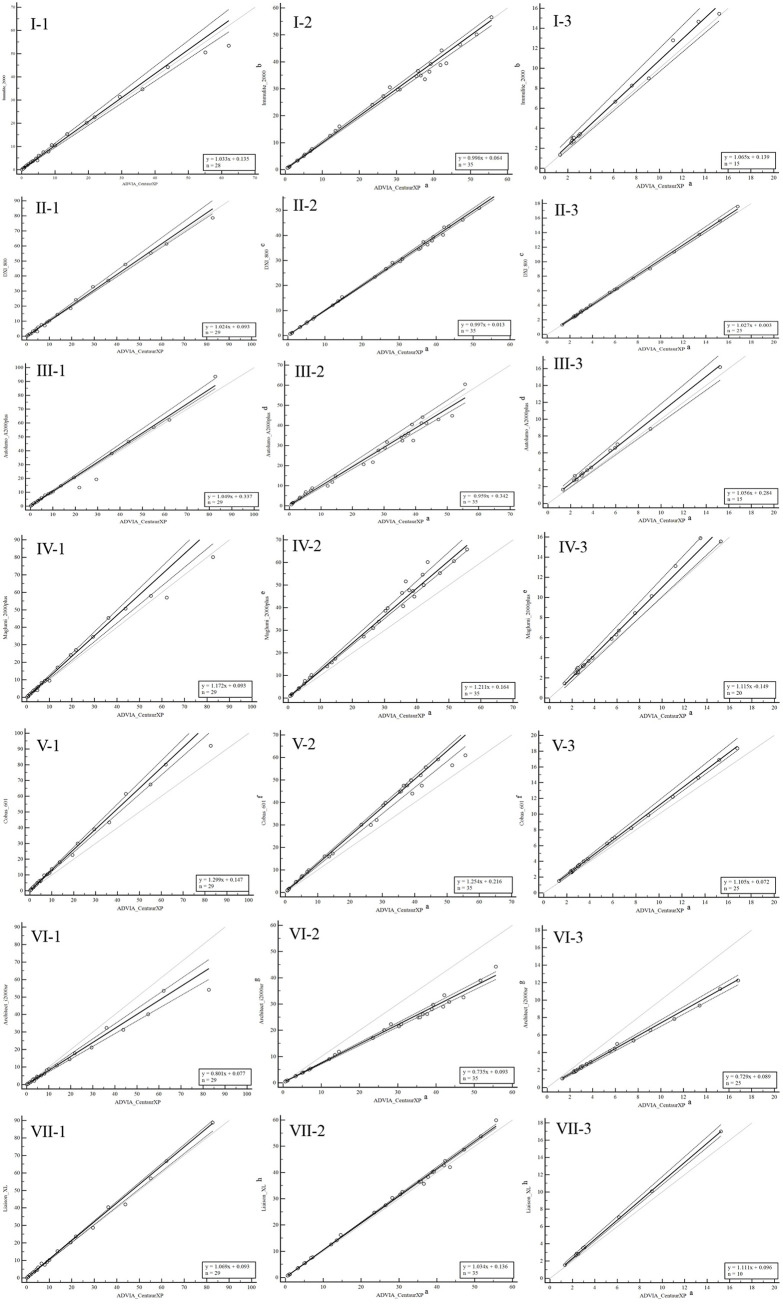
The comparability of thyroid-stimulating hormone (TSH) measurements when individual human serum samples (Left), non-commutable external quality assessment (EQA) materials (Middle), and commutable EQA materials (Right) were used after relevant corrections for systematic and commutability biases. a-h (Middle) and a-h (Right) denoted measuring systems listed in Tables 2 and 3, respectively.

The scatter plots for the assay pairs illustrating the linearity and the distribution of the data were presented in **Fig 5**.

## Discussion

TSH is currently the most sensitive and widely used marker of thyroid status in individuals with thyroid disease [4]. Nevertheless, although the functional sensitivity of TSH measurements has improved considerably with the introduction of “third”-generation systems, further studies are required to standardize these measurements [6, 16, 17].

Achievement of comparable results and standardization among different instrument platforms and measurement procedures has been anticipated by clinicians and laboratory personnel; however, these goals have yet to be realized over the mid-term, owing primarily to the lack of accepted reference measurement procedures for these measurements [17]. Furthermore, measurements were characterized by large systematic biases (-13.84%~9.85%). However, the correction of such systematic biases helped determine the actual relationships among immunoassays (**Table 1** and **Fig 2B**). Meanwhile, we identified similar relationships using NCCL EQA data after correcting for commutability-related biases (-42.26%~12.19%) (**Table 2, S5 Table,** and **Fig 2D**). For all data sources (a panel of individual serum samples, NCCL EQA data, or commutable EQA data), compared with the ADVIA CentaurXP assay, the Cobas 601 and Maglumi2000 plus assays yielded greater measurements and the Architect i2000sr assay provided lesser measurements (**Fig 5**), which tends to be consistent with certain previous findings [17, 18].

Even though commutability is one of the most important properties of a Proficiency Testing (PT)/EQA sample, most commercial PT/EQA samples are non-commutable owing to modifications in their preparation [10, 19]. The NCCL EQA materials used herein were commutable only in three or five of seven assay combinations (**Figs 3** and **4**) based on two different approaches. This relatively poor commutability in contrast with the findings of Clerico et al. [18], which could be explained on the basis of differences between the two studies with respect to the source of EQA materials and statistical methods used to assess commutability. Nevertheless, we believe that the merit of our study lies in the fact that we evaluated and corrected for commutability-related biases after correcting for systematic biases, which are common procedures used in routine laboratory practice. Accordingly, we observed similar relationships among immunoassays when using patient serum panel and pools and commercial EQA materials. In this study, we also described a method of harmonization using non-commutable EQA materials, which is important for EQA organization. Although we could not demonstrate the commutability of human serum pools (commutable EQA), some studies have provided evidence regarding the commutability of these materials through the same preparation technique [12, 14, 19, 20].

Nonetheless, this study has some notable limitations. First, we used EQA materials with only two concentrations of TSH to evaluate the systematic and commutability-related biases. However, for practical reasons, the evaluation of commutability of all lots of PT/EQA materials, which were obtained using the same preparation procedure, was unnecessary and not feasible [5]. Furthermore, systematic biases tended towards zero (**S5 Table**) and the relationships among immunoassays for NCCL EQA material were similar to those for patient samples (**Fig 5**), thereby providing evidence of the feasibility of this method. Second, owing to the insufficient volume of a sample obtained from one patient, we used only 29 samples to evaluate the comparability of TSH assays; However, using the ADVIA CentaurXP assay, the concentration range was well characterized from 0.076 μIU/mL to 82.625 μIU/mL. Third, we assessed only eight TSH immunoassays in this study; hence, future studies are required to verify the reliability of this method with a larger number of platforms.

In conclusion, this study shows systematic differences among the TSH immunoassay methods that were most widely used in China. Our data are potentially applicable to clinicians and experts in laboratory medicine to better compare and more correctly interpret patient results. The same relationships can also be clarified from EQA data, but only if the commutability-related biases are evaluated and corrected, which will make a valuable contribution to the harmonization and standardization of TSH measurements.

## Supporting information

S1 Table Analytical characteristics of the eight TSH immunoassays. (DOCX)

S2 Table The number of laboratories participating NCCL EQA (2016~2019). (DOCX)

S3 Table The number of laboratories participating commutable EQA (2017~2019). (DOCX)

S4 Table Systematic biases and commutability-related biases of NCCL EQA materials among 8 TSH immunoassays compared to ADVIA Centaur XP. (DOCX)

S5 Table Systematic biases and commutability-related biases of NCCL EQA materials among 8 TSH immunoassays compared to ADVIA CentaurXP after systematic biases were corrected. (DOCX)

S1 Data (XLSX)

## List of abbreviations

TSH: thyroid-stimulating hormone
EQA: external quality assessment
