# Supplementary material for: Comparability of thyroid-stimulating hormone immunoassays using fresh frozen human sera and external quality assessment data
Source: PLoS One. 2021 Jun 15;16(6):e0253324. doi: 10.1371/journal.pone.0253324 (PMC8205121; doi:10.1371/journal.pone.0253324)
Supplement: S1 Table — (DOCX) [file pone.0253324.s001.docx]

| Manufacturer | Platform | AMR (μIU/mL) | RR (μIU/mL) | Principle of detection | Cat. no. | Lot no. | Source of antibody | Traceability |
| --- | --- | --- | --- | --- | --- | --- | --- | --- |
| Siemens Healthineers (Tarrytown, NY) | ADVIA CentaurXP | 0.008-150 | 0.55-4.78 | CL | 6491080 | 114316 | monoclonal and polyclonal antibody | WHO 3rd IRP 81/565 |
| Siemens Healthineers (Gwynedd, UK) | Immulite 2000 | 0.004-75 | 0.4-4 | CL | L2KTS6 | 654 | monoclonal and polyclonal antibody | WHO 2nd IRP 80/558 |
| Beckman Coulter Inc. (Brea, CA) | DXI 800 | 0.015-100 | 0.34–5.60 | CL | 33820 | 724228 | monoclonal and polyclonal antibody | WHO 2nd IRP 80/558 |
| Autobio Diagnostics Co., Ltd.(Zhengzhou, China) | Autolumo A2000plus | 0.005-100 | 0.35-5.5 | CMIA | 30040803CM01 | 180312 | monoclonal antibody | WHO 3rd IRP 81/565 |
| Snibe Co., Ltd.(Shenzhen, China) | Maglumi 2000plus | 0.01-100 | 0.3-4.5 | CL | 130203001M | 00517122101 | monoclonal antibody | WHO 3rd IRP 81/565 |
| Roche Diagnostics GmbH (Mannheim, Germany) | Cobas 601 | 0.005-100 | 0.27-4.2 | ECL | 11731459 | 31103002 | monoclonal antibody | WHO 2nd IRP 80/558 |
| Abbott Diagnostics (Abbott Park, IL) | Architect i2000sr | 0.0025-100 | 0.35-4.94 | CMIA | 7K62-35 | 91052UI00 | monoclonal antibody | WHO 3rd IRP 81/565 |
| DiaSorin S.p.A (Saluggia, Italy) | Liaison XL | 0.004-100 | 0.3-3.6 | CL | 311211 | 193736 | monoclonal antibody | WHO 2nd IRP 80/558 |

**S1 Table. Analytical characteristics of the eight TSH immunoassays.**

AMR, analytical measurement range; RR, reference range; ECL, electrochemiluminescence; CL, chemiluminescence; CMIA, chemiluminesent microparticle immunoassay.
