# Supplementary material for: Comparability of thyroid-stimulating hormone immunoassays using fresh frozen human sera and external quality assessment data
Source: PLoS One. 2021 Jun 15;16(6):e0253324. doi: 10.1371/journal.pone.0253324 (PMC8205121; doi:10.1371/journal.pone.0253324)
Supplement: S2 Table — (DOCX) [file pone.0253324.s002.docx]

**S2 Table. The number of laboratories participating NCCL EQA (2016~2019).**

| Platform | sample no. | laboratory number | | | | | | |
| --- | --- | --- | --- | --- | --- | --- | --- | --- |
|  |  | 2016, first | 2016, Second | 2017, first | 2017, second | 2018, first | 2018, second | 2019, first |
| ADVIA CentaurXP^a^ | 35 | 290 | 280 | 298 | 302 | 340 | 335 | 365 |
| Immulite 2000^b^ | 35 | 18 | 17 | 22 | 16 | 17 | 18 | 15 |
| DXI800^c^ | 35 | 277 | 290 | 328 | 337 | 385 | 373 | 414 |
| Autolumo A2000 plus^d^ | 35 | 25 | 23 | 30 | 29 | 35 | 29 | 42 |
| Maglumi2000 plus^e^ | 35 | 29 | 31 | 36 | 36 | 50 | 51 | 68 |
| Cobas 601^f^ | 35 | 399 | 409 | 495 | 519 | 644 | 656 | 751 |
| Architect i2000sr^g^ | 35 | 186 | 189 | 209 | 209 | 252 | 246 | 272 |
| Liaison XL^h^ | 35 | 30 | 30 | 36 | 31 | 34 | 33 | 45 |

a, included Siemens Advia Centaur CP/XP; b, included Siemens Immulite 2000/2000 XPi; c, included Beckman DXI 600,DXI 800; d, included AutoLumo A2000/A2000 plus; e, included Snibe Maglumi 600/800/1000/1000Plus/2000/2000plus; f, included Roche Cobas e601/e602; g, included Abbott Architect i2000SR/i2000/i1000srP; h, included DiaSorin S.p.A LIALSON/XL.
