# Supplementary material for: Comparability of thyroid-stimulating hormone immunoassays using fresh frozen human sera and external quality assessment data
Source: PLoS One. 2021 Jun 15;16(6):e0253324. doi: 10.1371/journal.pone.0253324 (PMC8205121; doi:10.1371/journal.pone.0253324)
Supplement: S3 Table — (DOCX) [file pone.0253324.s003.docx]

**S3 Table. The number of laboratories participating commutable EQA (2017~2019).**

| Platform | sample no. | laboratory number | | | | |
| --- | --- | --- | --- | --- | --- | --- |
|  |  | 2017,first | 2017,second | 2018,first | 2018,second | 2019,first |
| ADVIA CentaurXP^a^ | 25 | 44 | 23 | 51 | 27 | 58 |
| Immulite 2000^b^ | 15 | 2 | 0 | 4 | 0 | 2 |
| DXI800^c^ | 25 | 53 | 19 | 65 | 23 | 80 |
| Autolumo A2000 plus^d^ | 15 | 0 | 0 | 5 | 2 | 5 |
| Maglumi2000 plus^e^ | 20 | 4 | 0 | 7 | 4 | 12 |
| Cobas 601^f^ | 25 | 87 | 34 | 100 | 25 | 140 |
| Architect i2000sr^g^ | 25 | 47 | 18 | 50 | 18 | 64 |
| Liaison XL^h^ | 10 | 0 | 0 | 3 | 0 | 2 |

a, included Siemens Advia Centaur CP/XP; b, included Siemens Immulite 2000/2000 XPi; c, included Beckman DXI 600,DXI 800; d, included AutoLumo A2000/A2000 plus; e, included Snibe Maglumi 600/800/1000/1000Plus/2000/2000plus; f, included Roche Cobas e601/e602; g, included Abbott Architect i2000SR/i2000/i1000srP; h, included DiaSorin S.p.A LIALSON/XL.
