# Supplementary material for: Comparability of thyroid-stimulating hormone immunoassays using fresh frozen human sera and external quality assessment data
Source: PLoS One. 2021 Jun 15;16(6):e0253324. doi: 10.1371/journal.pone.0253324 (PMC8205121; doi:10.1371/journal.pone.0253324)
Supplement: S4 Table — (DOCX) [file pone.0253324.s004.docx]

**S4 Table. Systematic biases and commutability-related biases of NCCL EQA materials among 8 TSH immunoassays compared to ADVIA Centaur XP.**

| Platform | systematic bias | | | Commutability-related bias | | |
| --- | --- | --- | --- | --- | --- | --- |
|  | 201811 | 201812 | mean | 201811 | 201812 | mean |
| ADVIA CentaurXP | 8.78 | 2.70 | 5.74 |  |  |  |
| Immulite 2000 | -3.53 | -6.41 | -4.97 | -1.76 | -0.88 | -1.32 |
| DXI800 | 0.14 | -5.30 | -2.58 | -7.76 | -5.01 | -6.39 |
| Autolumo A2000 Plus | -4.01 | -10.10 | -7.05 | -17.47 | -4.56 | -11.02 |
| Maglumi2000plus | -4.87 | -15.86 | -10.37 | -3.02 | -6.63 | -4.83 |
| Cobas 601 | -13.23 | -14.45 | -13.84 | -39.13 | -38.54 | -38.83 |
| Architect i2000sr | -2.46 | -3.55 | -3.01 | 13.08 | 24.21 | 18.65 |
| Liaison XL | 9.75 | 9.95 | 9.85 | 12.71 | 21.85 | 17.28 |
