# Supplementary material for: Comparability of thyroid-stimulating hormone immunoassays using fresh frozen human sera and external quality assessment data
Source: PLoS One. 2021 Jun 15;16(6):e0253324. doi: 10.1371/journal.pone.0253324 (PMC8205121; doi:10.1371/journal.pone.0253324)
Supplement: S5 Table — (DOCX) [file pone.0253324.s005.docx]

**S5 Table. Systematic biases and commutability-related biases of NCCL EQA materials among 8 TSH immunoassays compared to ADVIA CentaurXP after systematic biases were corrected.**

| Platform | systematic bias | | | Commutability-related bias | | |
| --- | --- | --- | --- | --- | --- | --- |
|  | 201811 | 201812 | mean | 201811 | 201812 | mean |
| ADVIA Centaur XP | 2.57 | -3.16 | -0.30 |  |  |  |
| Immulite 2000 | 1.42 | -1.60 | -0.09 | -7.00 | -6.16 | -6.58 |
| DXI800 | 2.72 | -2.85 | -0.07 | -12.85 | -10.25 | -11.55 |
| Autolumo A2000 Plus | 2.76 | -3.76 | -0.50 | -21.90 | -9.69 | -15.80 |
| Maglumi2000plus | 5.11 | -7.04 | -0.96 | -8.34 | -11.76 | -10.05 |
| Cobas 601 | -1.27 | -2.66 | -1.97 | -42.54 | -41.98 | -42.26 |
| Architect i2000sr | 0.48 | -0.65 | -0.09 | 6.93 | 17.45 | 12.19 |
| Liaison XL | -1.06 | -0.88 | -0.97 | 6.48 | 15.12 | 10.80 |
